# Effect of Continuous Audio Biofeedback During Postoperative Partial Weight Bearing in Older Patients—An Exploratory Study

**DOI:** 10.3390/jcm15145498

**Published:** 2026-07-14

**Authors:** Léa Staub, Arlene Vivienne von Aesch, Johannes Dominik Bastian, Heiner Baur

**Affiliations:** 1 School of Health Professions, Department of Physiotherapy, Bern University of Applied Sciences, 3008 Bern, Switzerland; 2 Department of Orthopaedic Surgery and Traumatology, Inselspital, University Hospital Bern, University of Bern, 3010 Bern, Switzerland

**Keywords:** partial weight bearing, continuous audio biofeedback, older individuals, physiotherapy, functional therapy

## Abstract

**Background/Objectives**: Adherence to partial weight-bearing prescriptions (PWBP) is challenging. The use of audio biofeedback (AB) can potentially help to better implement PWBP. This study aimed to investigate the effect of continuous AB provided by sensor insoles on partial weight-bearing load during functional tasks. **Methods**: Twenty older patients received a single AB training for PWBP management postoperatively. The prescribed limb load was measured (ground reaction force) during four activities (walking, walking with a 5 kg backpack, sitting–standing–sitting, and standing) with force–sensor insoles with continuous AB (n = 10) or without AB (n = 10). Individual deviation from the prescribed load and the influence of age and cognitive function were analyzed. **Results**: The intervention group (continuous AB) managed PWBP better for three out of four activities: The relative deviation was 104.4% ± 144.2 vs. 164.5% ± 164.5 for the 5 kg backpack walk, 57.6% ± 107.2 vs. 86.8% ± 122.4 for the sit–stand–sit task, and 8.0% ± 102.3 vs. 38.8% ± 131.7 for standing. For the 3 min walking activity, the relative deviation was 127.7% ± 121.9 vs. 116.0% ± 133.0 in favor of the control group. Mean differences were not statistically significant for any of the activities. **Conclusions**: After a single training session with continuous AB, PWBP can only be insufficiently met. Studies with multiple training sessions seem necessary to further test the potential of continuous AB in the management of PWBP.

## 1. Introduction

Lower limb fractures following trauma are highly prevalent among adults over 60 years of age and constitute a major economic burden on healthcare systems [1,2]. In many cases, partial weight bearing (PWB) is prescribed to optimize fracture healing, prevent mechanical overload that may compromise surgical outcomes, and reduce complications associated with prolonged immobilization, such as pneumonia, thromboembolism, or infections [2,3,4]. Current guidelines by the American Academy of Orthopedic Surgeons (AAOS) support the use of PWB in selected fracture cases [5], and evidence suggests that reduced mechanical loading can promote bone tissue regeneration [6,7].

Early postoperative mobilization, typically provided by physiotherapists, is critical for functional recovery in the older population. Failure to mobilize within 10 days after surgery is associated with markedly increased early mortality [3,8]. However, adherence to PWB instructions in individuals aged 60 years and older is limited [9]. This reduced compliance is multifactorial, involving difficulties in estimating the actual load applied to the operated limb, deficits in existing teaching methods [10], postoperative pain, age-related cognitive impairment, and reduced limb strength [7]. Research further shows that conventional teaching approaches insufficiently support accurate learning of PWB [11,12,13], and physiotherapists are generally unable to visually estimate weight bearing with acceptable accuracy, regardless of experience level [13].

The current clinical gold standard—training with a bathroom scale combined with verbal or tactile feedback—allows only static load assessment and is not transferable to dynamic, functional activities such as gait or transfers [2,14]. Alternative technologies, particularly audio biofeedback systems, offer more reliable dynamic assessment but are used infrequently due to cost considerations. Nevertheless, audio biofeedback devices have demonstrated superior validity and reliability for monitoring PWB during movement and are suitable for patients after lower limb fractures [10,11]. Continuous augmented feedback can enhance motor learning by facilitating both skill acquisition and refinement [15].

Among such technologies, in-shoe systems like the OpenGo Sensor Insole [16] allow real-time quantification of weight bearing during daily activities and provide immediate audio signals via a smartphone application [17]. Audio biofeedback has shown benefits in older adults with musculoskeletal disorders or mobility limitations, especially in contexts where overloading poses a risk [18,19,20]. A recent study by Müssig et al. suggests that audio biofeedback insoles can effectively support adherence to prescribed PWB [21]. However, no research to date has focused exclusively on populations over 60 years of age, despite their disproportionate vulnerability to postoperative complications and challenges in motor learning.

The primary aim of this study is to evaluate whether continuous audio biofeedback using OpenGo insoles during training of activities of daily living results in superior adherence to prescribed PWB compared with a single PWB instruction using audio biofeedback in adults over 60 years old. Due to short hospitalization, patients experience only short introduction sessions to PWB, which may not be suitable to adhere sufficiently to PWB instructions. A secondary aim is to determine whether cognitive impairment and advanced age influence PWB non-compliance. Based on existing evidence suggesting that older adults benefit from repetition and continuous external feedback, we hypothesize that in-shoe audio biofeedback will enhance adherence in this population. A final objective is to assess the feasibility and usability of the OpenGo Sensor Insole [16] as a potential instrument for future PWB instruction and monitoring.

## 2. Materials and Methods

### 2.1. Study Design

This study was conducted as an exploratory pilot study. As the focus was not primarily on feasibility outcomes, the study does not fully align with methodological recommendations for pilot studies [22]. The design followed the CONSORT extension for pilot trials. The Ethics Committee of the Canton of Bern deemed the project exempt from full ethical review and declared non-responsibility (BASEC Req 2021 01457). This “non-responsibility” means that the project, due to its pilot character, does not fall under the Swiss Human Research Act. The local federal ethics committee therefore does not require ethical approval. The study took place between July 2022 and January 2023.

### 2.2. Participants

Twenty postoperative patients were recruited from a regional hospital and randomly allocated to a control group (n = 10) or intervention group (n = 10). Since a priori sample size calculation was not possible, a convenience sample of n = 20 was chosen to assess feasibility aspects and potential preliminary effects that will help inform a future full-powered trial. Eligible individuals were ≥60 years old, had undergone lower limb surgery requiring PWB, and were able to ambulate independently before surgery. All participants provided written informed consent and spoke German or French. Exclusion criteria comprised severe cognitive impairment (MoCA < 10, see below), acute illness, neurological or other conditions limiting safe ambulation, and shoe sizes outside EU 36–45.

### 2.3. Data Collection Procedure

Demographic and anthropometric variables (age, gender, employment status, living situation, shoe size) were registered. Cognitive performance was assessed using the Montreal Cognitive Assessment (MoCA), given its high diagnostic accuracy in older adults and its relevance for adherence to rehabilitation instructions [7]. Impairment was categorized according to established thresholds, with severe impairment (MoCA < 10) leading to exclusion [23]. Lower limb loading during functional activities was recorded using the Moticon SCIENCE OpenGo Insole3 system (Moticon ReGo AG, Munich, Germany). The device has been validated for reliable measurement of vertical ground reaction forces and gait-related loading parameters [10,19]. Each insole integrates 16 capacitive pressure sensors, a 3D accelerometer, and a 3D gyroscope. Data were transmitted in real time via Bluetooth to the Moticon SCIENCE mobile application (Moticon’s analysis software for OpenGo insoles, v3.5) and stored on a secure digital (SD) card. Adjustable audio feedback thresholds enabled load-specific alerts. Insoles undergo factory calibration; user-specific calibration was not performed due to the requirement of full bilateral loading.

### 2.4. Intervention Protocol

All procedures were conducted in the hospital’s physiotherapy department. After completing the MoCA, participants’ insoles were replaced with appropriately sized OpenGo Insole3 devices. The surgeon-prescribed PWB limit was entered manually into the mobile application.

Participants were randomized via the “Randomiser for Clinical Trial Free^®^” app. Walking aids were checked and adjusted as needed. During the first postoperative mobilization session (one day prior to testing), participants received standard instruction in PWB using the bathroom scale method and practiced ambulation with their prescribed walking aid. They then familiarized themselves with the insoles by walking between parallel bars while the physiotherapist monitored load data.

Warm-up:

Both groups completed a ~10 min warm-up with audio biofeedback activated. Participants practiced gradually loading the operated limb until the beep indicated that the PWB threshold had been exceeded; this was repeated 10–15 times. The warm-up further included a 3 min walk and two sit–stand–sit repetitions. A 5 min rest before data collection minimized fatigue. Randomized group allocation to these two groups took place:

- Control group: audio feedback active only during warm-up; deactivated during measurements.
- Intervention group: audio feedback active throughout warm-up and all measurements.

Functional testing comprised the following tests that all participants completed:

- Sit–stand–sit (3 times)
- Standing with walking aid (1 min)
- Level walking (3 min)
- Level walking with 5 kg backpack (1 min)

The full protocol lasted ~45 min. Participants then provided written feedback on their user experience.

### 2.5. Data Processing

Insole data were analyzed using the OpenGo software (v3.5). The “Static Report” and “Gait Report” functions provided maximum and average ground reaction force values [N]. For gait cycles, the software calculated the mean of peak stance phase loads.

### 2.6. Outcomes and Analysis

The primary outcome was deviation from the surgeon-prescribed PWB. Ground reaction forces were recorded in Newtons [N] and converted to kilograms (10 N = 1 kg). Activity-specific metrics included:

- Sit–stand–sit: maximum total load
- Standing: mean total load
- Walking tasks: mean peak stance phase loads

If automatic step detection was insufficient, peaks were identified manually. Secondary outcomes examined the influence of age and MoCA score on PWB compliance, as well as feasibility indicators from participant feedback and device performance. Compliance was defined as loading ≤ prescribed PWB; overload was classified as non-compliance.

Statistical analyses were conducted using R^®^ (Version 3.6.0). Descriptive statistics were calculated to characterize the two groups and the number of participants adhering to the prescribed PWB. All weight-bearing data were converted from Newtons to kilograms (10 N = 1 kg) and expressed relative to participants’ body weight. Normality of anthropometric and outcome variables was assessed using the Shapiro–Wilk test. Between-group differences in relative deviation from the prescribed PWB were analyzed using two-sample t-tests, with statistical significance set at *p* < 0.05, and 95% confidence intervals were reported. PWB compliance was evaluated by comparing the deviation between measured load and the surgeon-defined PWB target. Loads equal to or below the prescribed limit were classified as compliant, whereas higher loads indicated non-compliance. The influence of age and cognitive function (MoCA score) on PWB compliance was examined using linear regression.

## 3. Results

### 3.1. Participants Characteristics

Initially, 29 patients were identified to be eligible for participation. Nine patients declined participation due to lack of interest (n = 4), insufficient time (n = 1), or excessive pain at the time of recruitment (n = 4). Finally, 20 participants were enrolled and completed the study. The final sample consisted of nine women and eleven men. Group characteristics (mean ± SD) are presented in Table 1. The mean age was 69.2 years, and mean body weight was 78.2 kg. Cognitive screening showed no impairment in either group (MoCA intervention: 27.7 ± 2.9; control: 28.2 ± 2.3). Participants underwent various lower limb surgeries, including ankle osteosynthesis (n = 6), hallux or metatarsal arthrodesis (n = 5), femoral procedures (n = 3), Achilles tendon reinsertion (n = 2), ankle arthroplasty (n = 1), and calcaneal refixation (n = 1). Sixteen participants used crutches, three used a rollator, and one used a walking frame. Prescribed PWB limits ranged from 10 to 30 kg, with most participants instructed to maintain 15 kg.

### 3.2. Partial Weight Bearing with Continuous Audio Biofeedback vs. Training-Only Audio Biofeedback

Shapiro–Wilk testing confirmed normally distributed data. As shown in Table 2, deviations from the prescribed PWB were consistently higher during dynamic tasks compared to static ones. No statistically significant differences were found between groups. During the 3 min walking task, the intervention group showed a relative deviation of 127.7% ± 121.9 compared with 116.0% ± 133.0 in the control group (*p* = 0.85).

**Figure 1:**
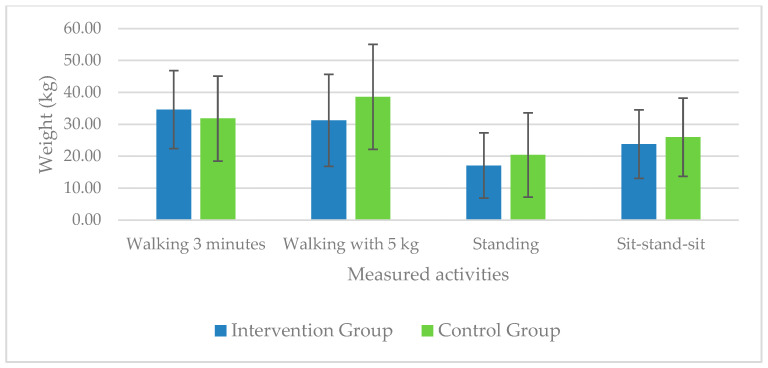
Weight-bearing load of the patient’s PWB leg for both groups and for each activity. Amount of the weight bearing in kg of the patient’s PWB leg during the measured activities for control and intervention groups. Data expressed as: mean of weight bearing on the PWB leg in kilogram with standard deviation (SD) represented in bar graphs.

When walking with a 5 kg backpack, deviations were 104.4% ± 144.2 in the intervention group and 164.5% ± 164.5 in the control group (*p* = 0.41). For standing, the deviation amounted to 8.0% ± 102.3 versus 38.8% ± 131.7 (*p* = 0.57), and for sit–stand–sit to 57.6% ± 107.2 versus 86.8% ± 122.4 (*p* = 0.58). Although these differences were not statistically significant, the intervention group demonstrated smaller deviations in three out of four tasks, especially in static conditions. Absolute load values ranged from 18 to 20 kg during standing and from 31 to 39 kg during backpack walking, as illustrated in Figure 1.

With respect to individual adherence, no participant met the prescribed PWB across all four activities. A proportion of participants adhered for certain tasks, with adherence rates presented in Table 2. During the 3 min walking task, adherence was slightly higher in the intervention group (13%) compared to the control group (11%). While walking with a 5 kg backpack, adherence was 11% in the intervention group, while none of the control participants adhered. For standing, 50% of participants in each group adhered, and for the sit–stand–sit task, 30% adhered in both groups.

### 3.3. Influence of Cognitive Level and Age on PWB Compliance

Linear regression analyses showed no significant association between cognitive level (MoCA score) or age and the amount of weight bearing in any of the measured activities, as summarized in Table 3.

### 3.4. Feasibility of the OpenGo^®^ System

One participant reported difficulties using the insoles with a lower leg cast, as the insole had to be placed inside a sock wrapped around the cast, which caused concerns about slipping. All other participants reported no noticeable differences while walking with the insoles. Audio biofeedback was generally perceived as helpful; several participants reported increased safety or a better understanding of how little or how much they were actually loading the operated limb. Participants in the control group occasionally felt uncertain about appropriate loading once audio feedback was deactivated. Two participants found it challenging to determine the correct PWB during static testing when asked to load the limb until the beep was heard. Overall, the OpenGo^®^ system was considered easy to use, and the supplier’s online manual was regarded as helpful for configuring audio thresholds and entering the prescribed PWB.

## 4. Discussion

The aim of this study was to examine whether continuous audio biofeedback delivered via sensor insoles improves adherence to prescribed partial weight bearing (PWB) during functional activities via a single bout of PWB introduction and exercise. Although adherence varied considerably across activities, continuous audio biofeedback did not result in statistically significant improvements compared with feedback provided only during initial instruction. Nonetheless, the intervention group demonstrated smaller deviations in three of the four assessed tasks, suggesting a potential but modest clinical benefit. Since adherence to PWB specification was low in both groups, the conclusion is that a single PWB instruction session is certainly not enough, and more practice sessions are needed for proper adherence. This seems valid for both feedback modes (initial audio feedback during training/continuous audio feedback during training and testing).

Participants showed distinctly greater deviations during dynamic, cyclic tasks than during static activities. This difficulty in maintaining PWB during gait is consistent with the higher motor complexity and the reduced ability to rely on fixed external support. During static tasks, participants could rely on the upper limbs and assistive devices, likely reducing inadvertent overload. The slight advantage observed in the control group for the 3 min walk lacks clinical relevance, given the non-significant statistical difference and the substantial measurement variability. Importantly, a system-inherent measurement error of approximately 10% must be considered when individual calibration is not possible, potentially contributing to increased variability and misclassification of borderline values.

The absence of statistically significant between-group differences may partly reflect the small sample size and high interindividual variability in loading behavior. These findings align with a recent study using the same insole system in adults aged 19–92 years, which showed that individuals over 65 years had more difficulty adhering to PWB despite audio biofeedback, although results were also not statistically significant [24]. Methodological aspects such as strict adherence definitions (any overload was classified as non-compliant) and the use of average peak loading as the outcome measure may further explain low adherence rates.

Other studies using audio biofeedback during PWB training reported beneficial effects but under different conditions. In healthy adults, training with audio biofeedback resulted in significant reductions in overload over repeated sessions, even without real-time feedback during measurement [10]. Hershko et al. demonstrated highly effective adherence in older adults when daily supervised PWB training with audio feedback was provided [6]. Similarly, Hustedt et al. found improved compliance when audio biofeedback was used consistently throughout rehabilitation; however, their younger sample and methodological limitations restrict comparability [25]. Collectively, these studies support the potential of audio biofeedback to reduce overload, even if overall adherence remains low. Low adherence to PWB has been widely reported, independent of age [18,26]. Studies suggest that the lower the prescribed PWB, the more challenging it is for patients to comply [24]. In our study, adherence was poor across all activities and groups, despite a widely used prescription of 15 kg PWB. Similar low adherence rates have been observed in patients prescribed 20 kg PWB postoperatively, even when audio biofeedback was available [18]. Interestingly, despite low compliance, re-fracture is rare, raising questions about whether very low PWB thresholds are clinically necessary in all cases.

Activity-specific adherence patterns also emerged. While many studies focus exclusively on walking, the present study analyzed non-cyclic functional tasks, revealing considerable variability between activities. Limited research has examined PWB compliance beyond gait, likely because many insole-based systems are optimized for cyclic movements and offer limited support for analyzing non-cyclic loading.

There is also no consensus regarding PWB prescription modalities: some authors recommend absolute loads (e.g., 15–20 kg), while others prescribe a percentage of body weight. No universal guideline exists for specific postoperative protocols, limiting comparability across studies. The approximate 15 kg PWB prescribed in our cohort is common in clinical practice but may not represent an optimal or evidence-based load prescription.

No significant associations were identified between age or cognitive performance and PWB compliance. This likely reflects the small sample size and the high average MoCA scores in our cohort, indicating normative cognitive functioning. Literature suggests that advanced age, cognitive deficits, low psychomotor skills, and reduced grip strength may negatively influence PWB adherence [7,9], but these effects could not be confirmed in this sample due to its homogeneity.

This study has several limitations that should be considered when interpreting the findings. First, the small sample size (n = 20) limits statistical power and increases the risk of type II error, which may partly explain the absence of statistically significant between-group differences despite observable trends. In addition, the exploratory pilot design restricts the generalizability of the results and precludes firm conclusions regarding effectiveness. Second, the intervention consisted of only a single training session, which is unlikely to reflect the learning processes required for sustained adherence to partial weight-bearing prescriptions—although this single session approach often corresponds to current clinical practice. Motor learning in older adults typically requires repeated practice and reinforcement; accordingly, previous studies have demonstrated that repeated or supervised biofeedback training leads to greater improvements in weight-bearing control compared with single-session interventions [27,28]. Third, the study population was relatively homogeneous, particularly with regard to cognitive function, as participants showed predominantly normal MoCA scores. This limits the ability to detect associations between cognitive impairment and weight-bearing compliance and reduces the applicability of the findings to more cognitively impaired populations. Fourth, heterogeneity in surgical procedures and prescribed weight-bearing limits introduces additional variability that may have influenced loading behavior. Previous research has shown considerable discrepancies between prescribed and actual weight bearing after surgery, particularly in the early postoperative phase, further contributing to interindividual variability [29]. Similarly, the use of different walking aids may have affected task performance and load distribution. Fifth, methodological aspects related to outcome measurement should be considered. The insole system, although validated, has an inherent measurement error (approximately 10%), particularly in the absence of individual calibration. Moreover, limitations in step detection and the need for occasional manual data extraction may have introduced measurement inaccuracies, especially during non-cyclic or short-duration loading phases. Sixth, the definition of non-compliance as any load exceeding the prescribed threshold may be overly stringent and could have led to an underestimation of clinically acceptable performance. In addition, the use of average peak loading values may not fully capture the variability and transient overloads occurring during functional activities, which have been highlighted as relevant factors in previous biofeedback studies [27]. Finally, the experimental setting may not fully reflect real-world clinical conditions. Participants performed standardized tasks in a controlled environment shortly after instruction, which may differ from habitual behavior during daily life. Evidence from controlled trials suggests that adherence patterns may differ substantially under supervised versus unsupervised or between no-feedback and feedback [28], emphasizing the need for ecologically valid study designs. Future studies should therefore include longer observation periods and real-world monitoring to better assess ecological validity.

The final objective was to assess the feasibility of the OpenGo^®^ Insole3 system in an older postoperative population. Participants generally reported positive experiences, particularly regarding audio biofeedback. From an investigator perspective, data acquisition was straightforward, but data processing proved more challenging due to limitations in step detection. As described previously [19], the system performs reliably during slow, continuous cyclic movements like walking, but the algorithm struggles with very short stance times—common when participants attempt to minimize loading. Because the software is not specifically designed for PWB monitoring, incomplete step detection occasionally required manual data extractions. Moreover, the mentioned lack of the possibility of individual calibration in a PWB setting reduces measurement accuracy. Finally, while the system represents a substantial financial investment (~10,000 CHF), it remains considerably more cost effective than installing force plates, making it a feasible tool for clinical settings.

## 5. Conclusions

This pilot study indicates that a single bout of PWB training with audio feedback does not lead to strong adherence to PWB recommendations during later monitoring without continuous feedback. Moreover, the single bout of PWB training with audio feedback plus the later continuous feedback during PWB seems to not be superior for PWB adherence. The OpenGo^®^ Insole3 system appears feasible for clinical use, particularly for gait-related monitoring, but current software limitations restrict accurate analysis of non-cyclic tasks. Importantly, the findings suggest that a single training session is insufficient to achieve reliable PWB performance irrespective of feedback mode. Continuous audio biofeedback should therefore be integrated into repeated, supervised training sessions to support motor learning and adherence. Future studies with larger samples should determine the optimal duration, frequency, and clinical benefit of such feedback-based PWB training protocols.

## Figures and Tables

**Table 1:** Characteristics of the control group and the intervention group.

Characteristic	Intervention Group n = 10	Control Group n = 10
Gender (female/male)	5/5	4/6
Age (years)	68.8 ± 9.6	69.5 ± 7.3
Weight (kg)	79.0 ± 22.7	78.7 ± 11.5
BMI (kg/m^2^)	25.6 ± 5.3	26.5 ± 3.0
MoCA Score ^1^	27.7 ± 2.9	28.2 ± 2.3
PWB leg (left/right)	6/4	6/4

Data expressed as: mean ± sd. Abbreviations: BMI Body Mass Index; MoCA Montreal Cognitive Assessment; PWB partial weight bearing; ^1^ no cognitive impairment > 25; mild cognitive impairment 18–25; moderate 17–10; severe < 10.

**Table 2:** Percentage of adherence at PWB for both groups according to activities.

Activity	Intervention Group ^1^	Control Group ^1^
Walking 3 min	13% 1/7	11% (1/8)
Walking with 5 kg	11% (1/8)	0% (0/10)
Standing	50% (5/5)	50% (5/5)
Sit–Stand–Sit	30% (3/7)	30% (3/7)

Data expressed as: percentage of adherence (%) (number with adherence/number without adherence). ^1^ non-usable data are not included in the total.

**Table 3:** Linear regression results for the influence of age and MoCA Score on the PWB.

Activity	MoCA Score	Age in Year
Walking 3 min	−1.72	0.61
	(0.287)	(0.226)
Walking with a 5 kg backpack	1.14	0.3
	(0.622)	(0.653)
Standing	0.84	−0.02
	(0.595)	(0.97)
Sit–stand–sit	−0.18	0.03
	(0.933)	(0.938)

Data expressed as: regression coefficient (*p*-value).

## Data Availability

Dataset available on request from the authors.
